# Supplementary material for: The delivery of creative socially prescribed activities for people with serious mental health needs during lockdown: Learning about remote, digital and hybrid delivery
Source: PLoS One. 2024 May 15;19(5):e0301550. doi: 10.1371/journal.pone.0301550 (PMC11095676; doi:10.1371/journal.pone.0301550)
Supplement: Attachment — Submitted filename: Response to Reviewers2.docx [file pone.0301550.s002.docx]

**Response to Reviewers**

Thank you for your additional guidance regarding the resubmission of this manuscript. We have incorporated the feedback and made changes as requested. Please find details of these changes documented below.

Comments to the Author

1. If the authors have adequately addressed your comments raised in a previous round of review and you feel that this manuscript is now acceptable for publication, you may indicate that here to bypass the “Comments to the Author” section, enter your conflict of interest statement in the “Confidential to Editor” section, and submit your "Accept" recommendation.

Reviewer #3: All comments have been addressed

2. Is the manuscript technically sound, and do the data support the conclusions?

The manuscript must describe a technically sound piece of scientific research with data that supports the conclusions. Experiments must have been conducted rigorously, with appropriate controls, replication, and sample sizes. The conclusions must be drawn appropriately based on the data presented.

Reviewer #3: Yes

3. Has the statistical analysis been performed appropriately and rigorously?

Reviewer #3: Yes

4. Have the authors made all data underlying the findings in their manuscript fully available?

The PLOS Data policy requires authors to make all data underlying the findings described in their manuscript fully available without restriction, with rare exception (please refer to the Data Availability Statement in the manuscript PDF file). The data should be provided as part of the manuscript or its supporting information, or deposited to a public repository. For example, in addition to summary statistics, the data points behind means, medians and variance measures should be available. If there are restrictions on publicly sharing data—e.g. participant privacy or use of data from a third party—those must be specified.

Reviewer #3: Yes

5. Is the manuscript presented in an intelligible fashion and written in standard English?

PLOS ONE does not copyedit accepted manuscripts, so the language in submitted articles must be clear, correct, and unambiguous. Any typographical or grammatical errors should be corrected at revision, so please note any specific errors here.

Reviewer #3: Yes

6. Review Comments to the Author

Please use the space provided to explain your answers to the questions above. You may also include additional comments for the author, including concerns about dual publication, research ethics, or publication ethics. (Please upload your review as an attachment if it exceeds 20,000 characters)

Reviewer #3: The article shares insight into how Voluntary Community and Social Enterprise (VCSE) organisations responded to the challenges of social distancing during the COVID-19 pandemic. The COVID-19 had multiple impacts on the human beings and overcoming it required various psycho-social measures. This article highlights some of it.

After careful reviewing, before we can recommend acceptance, we would kindly request you to address some minor outstanding concerns regarding methodology.

Specifically, we request that you update the manuscript to provide:

a) additional information regarding the participants in the interview study (ln 138-140), in addition to the gender information already provided, such as their age range and ethnicity information, which would not compromise their individual identities

- **We do not have age or ethnicity information.**

b) some examples of interview questions posed to study participants (ln 169 onwards)

- **The following examples have been added:**

During the interviews participants were asked to describe: the activities they provided prior to lockdown; how they adapted or changed these activities during lockdown; which on-line platforms they used; how they stayed connected with people who did not like using on-line platforms; the challenges encountered; and which practices they intended to continue using.

The additional information requested would improve the transparency of the methodology and offer readers a deeper understanding of the responses recorded from the participants in this study.
